# Common Chromosomal Fragile Sites—Conserved Failure Stories

**DOI:** 10.3390/genes9120580

**Published:** 2018-11-27

**Authors:** Vasileios Voutsinos, Sebastian H. N. Munk, Vibe H. Oestergaard

**Affiliations:** Department of Biology, University of Copenhagen, 2200 Copenhagen N, Denmark; vasileios.voutsilos@bio.ku.dk (V.V.); tzk640@alumni.ku.dk (S.H.N.M.)

**Keywords:** common chromosomal fragile sites, large genes, introns, mitosis, early replicating fragile sites, neuronal diversification

## Abstract

In order to pass on an intact copy of the genome during cell division, complete and faithful DNA replication is crucial. Yet, certain areas of the genome are intrinsically challenging to replicate, which manifests as high local mutation propensity. Such regions include trinucleotide repeat sequences, common chromosomal fragile sites (CFSs), and early replicating fragile sites (ERFSs). Despite their genomic instability CFSs are conserved, suggesting that they have a biological function. To shed light on the potential function of CFSs, this review summarizes the similarities and differences of the regions that challenge DNA replication with main focus on CFSs. Moreover, we review the mechanisms that operate when CFSs fail to complete replication before entry into mitosis. Finally, evolutionary perspectives and potential physiological roles of CFSs are discussed with emphasis on their potential role in neurogenesis.

## 1. Introduction

**Figure 1:**
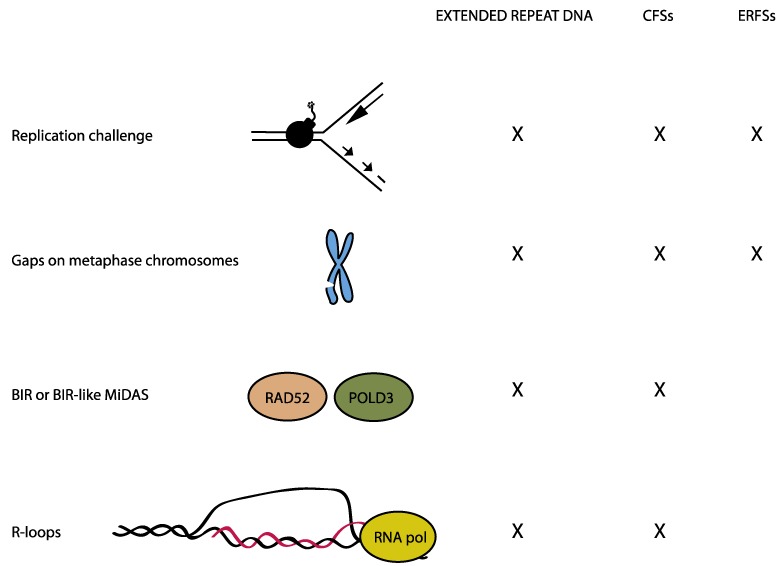
Similarities of three types of regions causing replication problems. Schematic summarizing similarities of expanded short tandem repeat sequence, common chromosomal fragile sites (CFSs), and early replicating fragile sites (ERFSs). See text for further details.

Regions that are difficult to replicate are at high risk of failing replication before cells enter mitosis, and this gives rise to microscopically visible gaps on metaphase chromosomes (Figure 1). Such regions, which are incompletely replicated in mitosis, are referred to as underreplicated. Several causes of local replication difficulties have been identified, including tandem repetitive DNA sequences, transcription activities, and timing of replication. Trinucleotide repeat expansions have been extensively characterized due to their involvement in diseases [1]. The challenge they pose to replication is intrinsic to the DNA sequence. However, studies of trinucleotide repeat expansion led to the discovery of another type of regions that are difficult to replicate due to certain transcription and replication patterns. These regions were dubbed **c**ommon chromosomal **f**ragile **s**ites (CFSs) [2]. They are late-replicating regions with large transcribed genes and they are fragile in all individuals. In this review, the large genes at CFSs are referred to as CFS genes. More recently, another type of fragile sites was discovered. These were called early replicating fragile sites (ERFSs), because they are replicated early in S phase and moreover, they are highly transcribed regions [3].

## 2. Short Tandem DNA Repeats

Some types of repetitive DNA sequences are comprised by short stretches of DNA that are repeated in tandem with a variable number of repeat units. More than a million short tandem DNA repeats (minisatellites and microsatellites, which includes trinucleotide repeats) are found in the human genome [4], and interestingly, many of these seem to be involved in the regulation of gene expression [5,6]. Short tandem DNA repeats have a tendency to diversify, which has been suggested as a means of genetic diversification with consequences for gene expression levels [7]. While the proposed physiological role of inherently unstable short tandem DNA repeats is relatively recent, pathological consequences of trinucleotide repeat expansions have been known for many years. Due to their involvement in diseases, trinucleotide DNA repeats have been widely studied and it is clear that this type of repeat DNA causes replication fork stalling and DNA damage [8]. Because short tandem DNA repeats cause replication problems, they are highly genetically unstable from generation to generation with extensive changes in copy numbers between individuals [9]. Therefore, short tandem DNA repeats are extensively used for forensic purposes [10]. Short tandem DNA repeats are also evolutionarily unstable as expected, and even at the somatic level, they vary in copy number [7,11].

The tendency of trinucleotide DNA repeats to increase in copy number is the underlying cause of a number of genetic diseases, collectively referred to as trinucleotide repeat expansion syndromes. These include Fragile X syndrome, Friedreich’s Ataxia, Huntington Disease, and many others, most of which affect the nervous system [7,12]. Fragile X syndrome was one of the first diseases found to result from repeat expansion on chromosome X [13,14], which causes the silencing of the gene *FMR1*. Moreover, alleles carrying the disease-causing repeat expansion at the *FMR1* locus were found to replicate later during the cell cycle [15]. Early studies of Fragile X revealed a correlation between Fragile X patients and microscopically visible gaps on the X chromosome in metaphase chromosome spreads from patient lymphoblasts, thus providing a cytogenetic feature that is useful for diagnosis and identification of carriers of Fragile X. However, conclusions that are based on cytogenetic analyses were initially uncertain because only a small fraction of the X chromosomes from male patients appeared as broken. 

Researchers thus explored culture conditions that would further induce gaps at the X chromosomes [16]. They found that when replication was challenged in cultured lymphoblasts, the fragility on the X chromosome became more evident in Fragile X patients. Taken together, these early studies hinted that replication difficulties at repeat expansions are a key problem. Indeed, many studies have confirmed that replication fork stalling frequently occurs at repeat DNA [1,17,18]. Short tandem repetitive DNA sequences, many of which can form secondary structures, such as hairpins and G-quadruplexes [19], may comprise obstacles to the replication machinery. At the same time, they have a tendency to form gaps on metaphase chromosomes in response to replication stress, which is why extensive trinucleotide repeat expansions give rise to so-called rare fragile sites that are occasionally associated with disease.

## 3. Common Chromosomal Fragile Sites

During the above-mentioned search for culture conditions that promoted Fragile X chromosome breakage, another important observation was made: lymphoblasts from healthy individuals also displayed recurrent chromosome gaps on metaphase chromosomes when they were cultured with low doses of the replication inhibitor aphidicolin (APH) (Figure 1) [20,21]. Regions that are susceptible to form gaps were named CFSs.

Years later, interest in CFSs was renewed when advanced array and sequencing techniques revealed that CFSs were highly mutated in a broad range of cancer genomes [22,23,24]. While cancer driver genes show a distinct pattern of enrichment for homozygous deletion, seminal work from Bignell et al. revealed that mutation patterns at CFSs were strikingly enriched for both hemizygous and homozygous deletions (with an average size of around 500 kb) across cancer genomes [22]. This reflects that whilst tumor suppressors are generally recessive, meaning that there is a selection for homozygous deletion during carcinogenesis, CFS deletions are bystander effects that are caused by their high mutation rates during conditions of replication stress. A caveat of this view is that haploinsufficient tumor suppressors may show enrichment for hemizygous deletions similar to fragile sites. Indeed, there are observations supporting that the fragile site gene *WWOX* is a haploinsufficient tumor suppressor [25]. Though certain CFSs seem to hold genes that may be tumor suppressors, it is still debated whether mutations at CFSs in general contribute to tumorigenesis [25].

Due to the shared features of CFSs with trinucleotide repeats (Figure 1), initial studies were focused on identifying sequence characteristics that would render CFSs prone to replication problems. This led to the finding that some CFSs are AT rich [26,27] and indeed the AT-rich regions of certain CFSs are difficult to replicate and they constitute hotspots for replication fork stalling [27,28,29]. However, the repertoire of CFSs is cell type-specific and varies between tissues showing that the fragility is not fully intrinsic to the DNA sequence [30,31,32]. Rather, CFSs coincide with large genes, the transcription of which is tightly linked to CFS breakage and their tendency to acquire deletions [33,34,35]. This is in agreement with the CFS characteristics that are derived from studies of cancer genomes [22,23,24].

## 4. Early Replicating Fragile Sites

Hydroxyurea (HU) is an inhibitor of the ribonucleotide reductase, thus HU treatment leads to the depletion of dNTPs. High doses of HU were found to result in DNA breaks at specific sites in the genome, called ERFSs [3]. As the name implies, ERFSs are located at early replicating regions of the genome. However, upon HU treatment followed by release into normal medium, the replication problem that is caused by HU persists throughout interphase and lead to the formation of cytogenetic gaps at ERFSs on metaphase chromosomes (Figure 1). ERFSs contain highly transcribed genes, the transcription of which contributes to their replication difficulties [3]. Recent work suggests that poly(dA:dT) tracts at ERFSs are responsible for replication initiation, yet they can also trigger replication fork collapse [36]. This was suggested to be due to the inefficient binding of RPA to poly(dA) sequence upon unwinding by the replicative helicase, leaving this uncoated strand vulnerable to nuclease attacks [36]. In avian DT40 cells, ERFS-like regions were identified based on their enrichment for the protein FANCD2, when cells were subjected to replication stress by APH treatment [33]. Similar to the previously described ERFSs, ERFS-like regions in DT40 contained dense clusters of highly transcribed genes that were replicated early in the S phase [33]. FANCD2 is a protein that is involved in the replication of CFSs as described below [28].

## 5. Transcription-Replication Conflicts

The transcription machinery may hinder the progression of the replication machinery since they work on the same template. Specifically, so-called R loops (Figure 1), where nascent RNA hybridizes with the DNA template and displaces the non-template strand can cause replication fork stalling and DNA double strand breaks (DSBs) [37,38,39]. Two types of transcription-replication conflicts (TRCs) can occur, namely co-directional (the two processes travel in same direction) and head-on (the two processes have opposite directionality). Both types cause replication problems but head-on collisions are more deleterious [40] and the cellular response is also different [38]. Using an episomal plasmid with a strong R-loop seeding sequence, it was found that head-on collisions exacerbate R-loop formation and trigger the ATR kinase that is normally associated with the replication stress response, whereas co-directional collisions alleviate R-loop formation and activates the ATM kinase known for its role in response to DSB [38]. Whilst TRCs that are caused by R loops are likely to be involved in CFS breakage in mitosis [28,41,42], it is more uncertain whether TRCs are direct causes of replication problems at ERFSs and expanded nucleotide repeats. 

Transcription at ERFSs is certainly contributing to their fragility [3], suggesting that TRCs directly induce replication problems at ERFS. Alternatively, transcription at ERFSs indirectly influences the coinciding fragility since transcription in G1 phase shapes the replication program in the following S phase [43]. Specifically, transcription activity in G1 clears transcribed regions of origin complexes from which replication is initiated [36,44]. Thus, the transcription profile of a cell is very important for determining the timing of replication. Likewise, the duration of G1 is crucial, because if G1 is shortened, full clearance of replication origins from long intragenic regions may not be accomplished, which may trigger TRCs during the S phase [43]. 

R loops often form at expanded trinucleotide repeats due to secondary structure formation on one strand, leaving the other strand susceptible to annealing with nascent RNA. Such R loops at the trinucleotide repeats contribute to repeat instability [45,46]. One well-characterized effect of R loops on repeat stability involves single-strand break formation, followed by base excision repair [45]. Another recent study finds that R loops at a trinucleotide repeat triggers socalled break-induced replication (BIR), and this mechanism underlies repeat expansion [47]. 

## 6. Transcription Timing of Common Chromosomal Fragile Sites

Transcription activity is evidently regulated to ensure that transcription and replication are kept separate [48,49]. In *Drosophila melanogaster,* active genes are replicated predominantly in early S phase, indicating a regulation of transcription timing in higher eukaryotes [50]. Similarly, one study found a spatial separation of replication from transcription in mouse cells [49], while another study established a temporal separation of the two cellular processes [48]. In fact, by utilizing a nascent RNA capture assay to map the transcriptional events throughout the S phase, transcription activity at a given time and place was found to inversely correlate to those of replication [48]. The spatiotemporal separation between replication and transcription serves to reduce conflicts between the two machineries. In the case of CFS genes, however, it is argued that TRCs are inevitable, since their transcription can span more than one cell cycle [41]. By performing quantitative reverse transcriptase PCR (RT-qPCR) on introns of CFS genes Helmrich et al. characterized the nascent expression of those genes in relation to the cell cycle phase. Surprisingly, they found that the transcription of at least some of the CFS genes start at the G2 or M phase, continues throughout the next cell cycle and it is completed in the G1 or early S phase in the cell cycle after that [41]. Such a transcription pattern makes TRCs inevitable, since the transcription of the genes will definitely coincide with the replication of the CFSs [41]. Replication of CFSs takes place late in S phase and replication stress can further delay replication with the result that CFSs go into G2 and M phase without being fully replicated [33,51,52]. The replication challenges are also caused by a scarcity of origins at CFSs [53]. After the induction of mild replication stress, increased rates of chromosome breakage are seen in CFS genes that are actively transcribed [41]. Interestingly, the hotspots for breakage are located at the areas that are transcribed during the S phase of the cell, which again indicates that TRCs are responsible for the CFS fragility [41]. R loops are also elevated at CFSs after APH treatment, which may correlate with increased incidence of TRCs in the CFS genomic region [41]. It is thus apparent that the transcription of CFS genes very often collides with the replication of the CFSs, which creates genomic instability.

## 7. Early Replicating Fragile Sites and Common Chromosomal Fragile Sites Comprise Underreplicated Regions in Mitosis

Collectively, it seems that transcription at ERFSs and CFSs, either directly or indirectly, increases the risk of cells entering mitosis prematurely before replication has been completed at these sites [3,33,34,42]. Yet, it is unclear whether the type of replication impediments that occurs at ERFSs or CFSs are intrinsically prone to escape checkpoint detection. Recent work from Lemmens et al. suggests that the replication process itself is inhibiting the activation of the mitotic kinases PLK1 and CDK1 via CHK1 [54]. However, a few ongoing replication forks may not be sufficient to repress PLK1 and CDK1. Also, work from the Cimprich lab shows that ATR activity relays ongoing replication to suppress the transcription of a set of mitosis-promoting genes in the primary RPE1 cell line [55]. This is in agreement with previous studies in cancer cell lines, which also found that ATR is key for suppressing mitosis until replication is complete [56,57]. Underreplicated genomic regions per se are not sufficient to suppress mitotic entry [58]. Rather, it seems that the active replication machinery signals via ATR and CHK1 to ensure the complete replication before mitotic onset. Consistently, both ERFS and CFS stability is dependent on ATR [3,59], but it remains unclear whether their tendency to escape checkpoint activation is linked to their transcription or other specific features.

## 8. The Consequences of Underreplicated DNA

### 8.1. FANCD2 and Anaphase Bridges

Research from the last decade has given important insight into the cell biology of CFSs by deciphering cellular consequences of replication problems at CFSs. It is now more clear how underreplicated CFSs manifest and how they are processed in mitosis and beyond. The DNA damage bypass mechanism called translesion synthesis may have a role in completing replication of CFSs [60,61]. This backup pathway probably works in G2 before mitosis [62]. If the replication problem at a CFS persists into mitosis, it can be visualized as so-called FANCD2 sister foci at the CFS on each sister chromatid [63,64]. FANCD2 is a key component of the Fanconi Anemia repair pathway, which provides cellular resistance to DNA cross-linking agents [65]. Similar to cross link-induced FANCD2 foci, FANCD2 foci at CFSs on metaphase chromosomes colocalize with the FANCD2 binding partner FANCI and they are dependent on FANCD2 monoubiquitylation by the Fanconi Anemia core complex [63,64].

FANCD2 foci in the M phase are thought to mark regions that have not been fully replicated before cells enter mitosis and chromatin immunoprecipitation sequencing (ChIP-seq) of FANCD2 in APH-treated cells consistently identifies CFSs [33,66]. In agreement with the view that gaps on metaphase chromosomes are the consequence of incomplete replication, G2-M checkpoint deficiency provokes and exacerbates replication stress-induced gaps at CFSs on metaphase chromosomes. Specifically, ATR, CHK1, and BRCA1 via their involvement in the G2-M checkpoint are important to prevent CFS breakage [59,67,68]. Notably, FANCD2 foci were observed both at broken and unbroken CFSs on chromosomes in metaphase. Upon progression into anaphase, FANCD2 foci segregate symmetrically on the sister chromosomes [63,64]. Interestingly, so-called ultrafine anaphase bridges (UFBs) often form between FANCD2 sister foci on anaphase chromosomes. UFBs are threads of DAPI-negative DNA interconnecting sister centromeres during normal anaphase [69], whereas replication stress specifically induces UFBs at CFSs [63,64]. The proteins PICH and BLM coat all types of UFBs [69], whereas FANCD2 defines the UFBs from CFSs and most often localizes to the termini of UFBs [63,64]. The recruitment of BLM to replication stress-induced UFBs is compromised in FANCA and FANCC deficient cell lines, whereas PICH recruitment is not affected by FANC deficiency [64,70].

The recruitment mechanism as well as the exact role of FANCD2 at CFSs in mitosis is somewhat obscure. FANCD2 does not seem to be required for recruitment of endonucleases (described below) to underreplicated CFSs in mitosis [70]. However, recent work from Madireddy et al. shows that FANCD2 is required for replication through an AT-rich region in the core of the most fragile region of FRA16D, which houses the *WWOX* gene [28]. Moreover, it was found that FANCD2 deficiency results in a marked increase in RNA-DNA hybrid formation at FRA16D and that the overexpression of RNaseH1 (an enzyme that is capable of degrading RNA in RNA-DNA hybrids) restores replication progression through FRA16D, collectively suggesting that FANCD2 works to facilitate replication through RNA-DNA hybrids at CFSs [28]. FANCD2 has a well-characterized role in inter-strand cross-link repair, which depends on its monoubiquitylation. In contrast, the role of FANCD2 in the replication of CFSs is not fully dependent on its monoubiquitylation [28]. Interestingly, BRCA2/FANCD1, which normally works downstream of FANCD2 monoubiquitylation, has a role in the replication of FRA16D that is similar to FANCD2 [28].

### 8.2. Endonucleases Promote Common Chromosomal Fragile Sites Breakage and DNA Synthesis in Mitosis

A number of structure selective endonucleases, including MUS81 and its binding partners EME1 and XPF-ERCC1, as well as the nuclease scaffold SLX4, localize to CFSs in mitosis where they are squeezed in between sister FANCD2 foci [70,71]. Here, the nuclease activity of MUS81 is promoting DNA synthesis in mitosis (referred to as MiDAS [72]) [73]. MUS81 activity is thought to create DNA strand breaks that are used to initiate DNA synthesis in a process that requires RAD52 but not RAD51 [72]. Similar to the previously characterized DNA synthesis pathway named BIR [74,75], DNA synthesis in mitosis depends on the non-catalytic subunit of polymerase delta called POLD3 (Figure 1) [73]. The cleavage activity of MUS81 is stimulated by the helicase RECQL5 and it was suggested that RECQL5 removes RAD51 from stalled forks, making them accessible to MUS81. Interestingly, this function of RECQL5 is dependent on Ser727 phosphorylation (in human RECQL5) by CDK1 [76]. CDK-mediated phosphorylation also promotes the complex formation of SLX4-SLX1 with MUS81-EME1 at mitotic onset [77]. Thus, the microscopically visible gaps on human metaphase chromosomes are actively generated by a process that requires MUS81 [70,71], and some of the gaps probably represent newly synthesized DNA at the mitotic chromosomes [70,71,73]. Interestingly, the recruitment of SLX4 to mitotic chromosomes depends on TopBP1 in avian DT40 cells [78]. Consistently, TopBP1 is found at APH-induced gaps on metaphase chromosomes and certain UFBs. TopBP1 also facilitates DNA synthesis in mitosis [78,79]. TopBP1 interacts with SLX4 in a manner that depends on Thr1260 (in human SLX4). This residue lies within a conserved motif, where the homologous motif in yeast SLX4 was shown to undergo CDK1-dependent phosphorylation, which stimulated the interaction between the yeast TopBP1 homolog Dpb11 and SLX4 specifically in mitosis [80]. 

Preliminary work that was published on BioRxiv describes a process that may work as an alternative to MiDAS for processing of underreplicated regions in mitosis [81]. This work suggests that the E3 ubiquitin ligase TRAIP mediates the unloading of the replicative helicase when cells enter mitosis and this unloading promotes error-prone repair of underreplicated regions by either RAD52- or Polθ-mediated annealing [81]. This model is consistent with the frequent deletions and sister chromatid exchanges that were observed at CFSs [22,82].

### 8.3. Consequences of Underreplicated Regions in G1 Daughter Cells

Underreplicated regions that persist in anaphase can lead to non-disjunction manifesting as either UFBs or chromatin bridges. UFBs may still be resolved in anaphase or telophase but they can leave a scar marked by large 53BP1 foci in subsequent G1 [83]. These are named 53BP1 nuclear bodies (53BP1 NBs) [83,84]. The 53BP1 NBs in G1 colocalize with γH2AX suggesting that they mark DNA damage. When cells enter S phase, 53BP1 NBs dissolve. This may reflect that they require homologous recombination-mediated repair rather than non-homologous endjoining (NHEJ), which is the favored pathway for repair in G1 [85]. The presence of 53BP1 NBs in G1 cells leads to extension of the G1 phase or entry in to a quiescent state in a manner that depends on p53 [86,87,88]. 53BP1 NBs also colocalize with so-called OPT-domains that contain the transcription factors Oct-1 and PTF, as well as the RNA helicase DDX1 [84], but transcription is repressed in 53BP1 NBs [84]. Importantly, other types of DNA stress that do not manifest as DNA bridges contribute substantially to 53BP1 NBs [78]. Thus, TopBP1 foci that persist throughout mitosis colocalize with 53BP1 NBs in G1, showing that TopBP1 constitutes a useful marker for DNA structures that precipitate DNA damage in daughter cells [62,78]. Deficiency in mitotic DNA processing also leads to missegregation, which in turn can cause binucleation [78] or the formation of micronuclei [63,64]. Micronuclei can trigger the highly mutagenic event chromothripsis [89].

## 9. Conserved Features of Common Chromosomal Fragile Sites Genes

Despite being highly unstable genomic regions, increasing evidence suggests that CFSs may hold conserved biological functions. Molecular mapping revealed that CFSs are conserved between mammals and birds and coincide with orthologous genes [33] and comparison of CFS fragility levels have shown that CFS fragility is conserved between mouse and human in homologous regions in lymphocytes [35]. Genes hosting CFSs are extremely large and they consist mainly of very long intronic sequences, which have persisted during evolution despite rendering the genes susceptible to genomic instability [33]. Analysis of gene lengths in 203 vertebrates revealed that large gene bodies of extremely long genes are conserved between most vertebrates [33]. Furthermore, while a global tendency of intron size reduction is observed in bird genomes, large introns in all CFS genes investigated (*PRKN*, *MARCOD2*, *GRID1*, *CCSER1*, *SETBP1*, *IMMP2L,* and *DACH1*) have to some extent resisted size reduction [33], supporting that large introns in CFS genes may hold a conserved biological function. 

The role of introns in creating diverse and complex transcriptomes through alternative splicing is well established. In addition, a recent study by Bonnet et al. proposes a role of introns in preventing R-loop accumulation and the associated genomic instability [90]. Genome-wide mapping of R loops and DNA-damage histone markers in budding yeast and human cell lines showed that among highly transcribed genes, genes without introns are more prone to form R loops and accumulate DNA damage than intron-containing genes. In budding yeast, intron deletion from highly transcribed genes led to de novo formation of R loops at the loci. Intriguingly, spliceosome assembly on intron-containing precursor messenger RNA (pre-mRNA) suppressed R-loop formation while insertion of a group I self-splicing intron failed to do so, suggesting that the recruitment of splicing factors to small introns prevent the formation of R loops rather than splicing per se. In contrast to this proposed role of small introns in budding yeast, it is evident that the extremely long introns in CFS genes promote conflicts between transcription and replication processes, including R loops rather than protect against them [28,41], which may be due to differences in the protein composition of small versus long introns in nascent pre-mRNA.

Altogether, while this recent study proposes a role of small introns in maintaining genome integrity of transcribed loci, the role of long introns in CFS genes seems to be associated with the coinciding fragility. In line with this, the extreme length of fragile site genes may generate extensive topological problems because negative supercoils build up in the DNA behind RNA pol II [91]. This phenomenon is thought to underlie R-loop formation at long genes, which occurs in response to the depletion of Top1, the topoisomerase responsible for relief of topological stress in DNA [92].

The fragility of CFSs may carry a functional significance [93]. As previously suggested, DNA breaks at CFSs may serve to activate the DNA damage checkpoint and induce apoptosis or senescence, thus constituting a barrier to transformation, as proposed for oncogene-induced replication stress [93,94].

**Figure 2:**
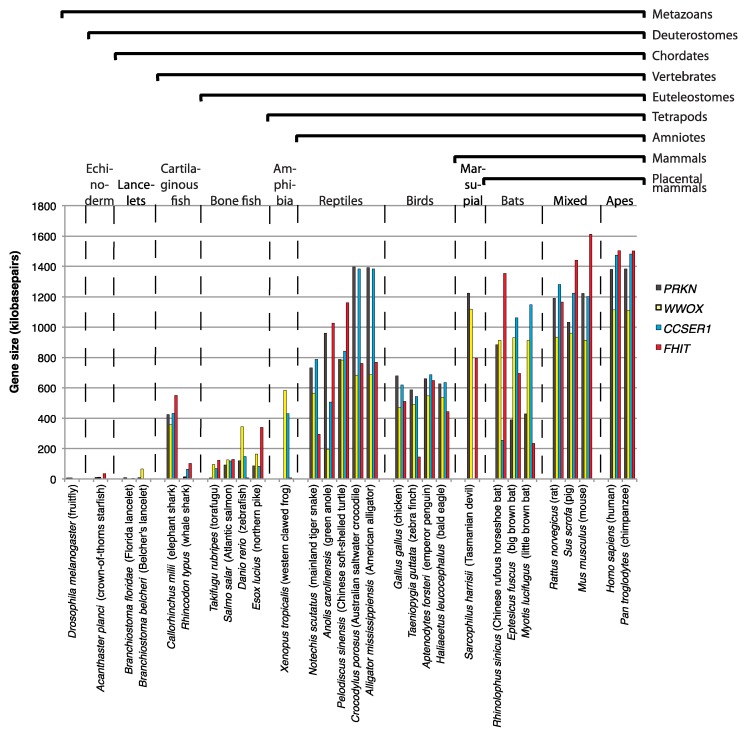
Bar chart showing gene sizes of the CFS genes *PRKN*, *WWOX*, *CCSER1*, and *FHIT* in various metazoan species with focus on vertebrates. Latin name and common name is given below the bars. Phylogenetic classification is indicated above the chart. Gene sizes are retrieved from the National Center for Biotechnology Information (NCBI) genes database.

To understand at which point during the evolution the vast size of CFS genes arose, we performed a simple analysis of CFS orthologue length in their phylogenetic context (Figure 2). We here focused on four well-studied CFS genes, *PRKN*, *CCSER1*, *WWOX,* and *FHIT*, where two of these, *PRKN* and *CCSER1*, were confirmed as conserved CFSs by their fragility in chicken DT40 cells [33]. When total gene length is plotted in selected grouped animals it is clear that the CFS genes under investigation started their expansion in an early vertebrate ancestor (Figure 2). Curiously, one of the represented cartilaginous fish, the elephant shark has larger CFS genes than any of the bone fish represented. Unfortunately, the *PRKN*, *CCSER1*, *WWOX,* and *FHIT* are poorly annotated in amphibian but based on the sizes of *WWOX* and *CCSER1*, which are represented it seems that they gain extra length in amphibians and certainly in reptiles, where the crocodile and alligator genomes present with *PRKN* and *CCSER1* genes that exceed 1.3 megabases in length. Birds have undergone a reduction in total genome size during evolution, which reflects an adaption to flight [95]. Thus, in general introns as well as intergenic regions are reduced in size in birds. However, our recent work showed that *PRKN* and *CCSER1* to some degree have escaped the reduction in intron sizes, suggesting that the extreme size of the genes may provide fitness to the organisms [33]. Mammals in general seem to have retained the extreme size of CFS genes although there are some exceptions in bats. Similar to birds, bat genomes have been reduced during evolution, which similar to birds is thought to be an adaption to flight. Thus, bats have the most compact of mammalian genomes. The tendency for reduced size is also reflected in the slightly smaller size of the CFS genes in bats, although it may not be proportional to the overall degree of genome reduction. Finally, we note that some of the inconsistencies within groups are likely due to annotation errors.

## 10. Neuronal Genetic Diversity Caused by Common Chromosomal Fragile Sites

Since CFSs seem to be detrimental, or at best neutral for the cells, why are they retained throughout evolution? A proposed explanation suggests that CFSs may have a role in producing the immense diversity [96,97] that has been observed in the brain cells [98,99]. CFSs are particularly fragile when subjected to replication stress and this can lead to genomic structural variations [34,100,101], which in turn can be part of the underlying cause for the diversity of the neurons. In agreement with this hypothesis, single cell sequencing of the human frontal cortex has shown that 13–41% of its neurons contain at least one mega-base scale copy number variation [101]. Moreover, the condition of replication stress for structural variations to arise is met as neural stem cells undergo massive rapid proliferation during neurogenesis generating replication stress [102]. This is also evident by the fact that DNA repair factors that are implicated in replication stress-associated damage are critical for proper neurodevelopment. One example of this is the ATR-Seckel syndrome that is a result of ATR deficiency. The ATR-Seckel syndrome is largely characterized by defects in the nervous system [103]. TopBP1 is another DNA repair factor deficiency of which results in the ablation of the structure of the cortex in a p53 dependent manner [104]. It has also been shown that neural proliferation continues in adult brains as well, particularly in the subventricular zone and subgranular zone [105,106]. In fact, approximately 700 new neurons per day are generated in the hippocampus. This corresponds to 1.75% annual turnover of the neurons in the renewing fraction [107]. Such proliferation implies that replication stress could still occur in the adult brain and its consequences could be aggravated given that the availability of dormant replication origins on the genome diminish in neural stem progenitor cells (NSPCs) as compared to embryonic stem cells (ESCs) [108].

In a recent study by Wei et al. recurrent DSB clusters (RDCs) occurring in NSPCs of mice were identified using high throughput genome-wide translocation sequencing (HTGTS) [96]. Using stringent criteria they found 27 RDCs. These were mostly located on transcriptionally active, large (>100 kb), late-replicating genes, which are all characteristics that are associated with CFSs and six out of the 27 RDCs identified in the study correspond to known CFSs in the human genome [96]. Importantly, most of those RDCs could only be detected after induction of mild replication stress with APH. Remarkably, the RDCs are all associated with genes that are implicated in neural processes and disorders [96]. Wei et al. propose that DSBs may at least partially explain the CNVs that were observed in the human brain. Thus, CFSs may contribute to the genetic diversity of the neurons.

## 11. A potential Link between Common Chromosomal Fragile Sites and LINE1-Dependent Neuronal Genetic Diversification

CFSs might also have a supportive role in retrotransposon LINE1-dependent diversification of the neuronal genomes [109,110]. LINE1 retrotransposons have been shown to influence neuronal cell differentiation/maturation in cell culture and cause neuronal mosaicism in the brain of mice [111]. Furthermore, some LINE1 retrotransposons can be expressed as a result of the Wnt singalling pathway in the same way that the proneurogenic factor NEUROD1 is expressed, indicating a possible role of LINE1 in neurogenesis [112]. Although, LINE1 seems to have an important role in neurogenesis, its integration process can induce genomic instability [113]. It was shown that LINE1 expression could cause a large number of DSBs, as indicated by increased γ-H2AX foci formation. The number of DSBs is greater than the predicted number of successful integrations of LINE1, indicating that the LINE1 integration process is largely inefficient [114]. According to a recent study, replication stress that results from expression of long neural genes renders glioblastoma cancer stem-like cells radiation resistant [115]. The group proposes that this is because of the constitutive activation of the DNA damage response, as a result of the replication stress at the long neural genes. When active, the DNA damage response can in turn facilitate DSB repair that is caused by radiation, thus conferring resistance to radiation [115]. Following this line of logic, it is conceivable that CFSs hosting long neural genes and the replication stress that they generate when transcribed act as a minor challenge that keeps the neural cell prepared for the major physiological challenge that is imposed by LINE1 retro transposition. CFS rearrangements may distinctly promote neuronal diversification through both direct and indirect mechanisms.

## 12. Potential Neuronal Epigenetic Diversity Caused by Common Chromosomal Fragile Sites

**Figure 3:**
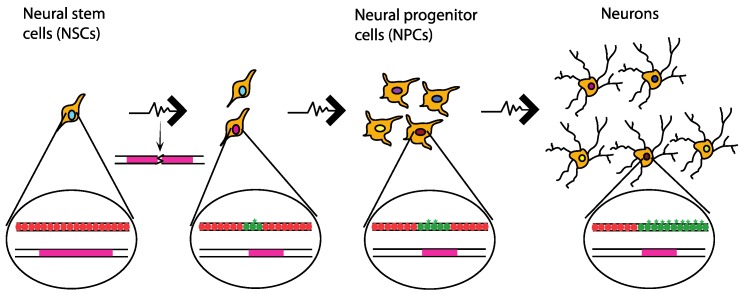
Hypothetical model showing diversification of neurons as a result of replication stress at CFSs. As neural stem cells (NSCs) proliferate, replication stress (spiked arrow) can result in loss of coordination of the histone deposition and replication process. As a consequence, histones that carry modifications (red circles) are replaced by newly synthesized unmodified histones (green circles). Replication stress at CFSs also causes accumulation of the histone variant macroH2A1.2 (green star). At the genetic level structural variations, like deletions, can arise due to replication stress-induced breaks. The variance at the genetic and the epigenetic level that is caused by replication stress throughout the differentiation of neurons can lead to variance of the expression of the genes located at CFSs. Each individual cell will then have a different expression profile (as indicated by the different colours of cell nuclei), which will eventually lead to a diverse population of neurons that is necessary to perform the complex functions of the brain.

Genetic diversity is not the only potential explanation of the mosaicism observed in the brain. Such diversity could also stem from epigenetic diversification of the neurons. In particular, replication stress in primary fibroblasts can cause the accumulation of the histone variant macroH2A1.2 at CFSs in a FACT-dependent manner [116]. MacroH2A1.2 facilitates homologous recombination-mediated DNA repair by recruiting the recombination factor BRCA1 [116] and it has been associated with H3K27me3-containing chromatin and senescence-associated heterochromatin [117,118]. Similarly, Papadopoulou et al. found replication stress-induced changes of gene expression due to epigenetic changes in a region of the genome that forms G4 quadruplex structures, which complicates replication of the region [119]. In fact, expression levels of the BU-1 gene, which contains a G4 quadruplex structure, are reduced when replication stress is induced by APH or HU treatment. The reduction in expression is interpreted as a consequence of the lack of coordination of replication and histone deposition processes, due to the delayed replication that is caused by the G4 structure and aggravated by the aforementioned drug treatments [119]. It is therefore tempting to speculate that the gradual accumulation of epigenetic changes in CFSs owed to replication stress [116] can cause alterations in the expression of the CFS genes, which are largely associated with neural related functions [96,120] and therefore lead to cell diversification of the neurons (Figure 3).

## 13. Conclusions and Perspectives

CFSs are regions prone to TRCs, replication stress and breakage as well as sources of increased genomic instability. Nevertheless, they, along with their characteristics, seem to be conserved among vertebrates. The large size of CFS genes somehow has resisted shortening throughout the evolutionary timeline, despite the fact that it contributes to their instability. Their conservation seems even more surprising when considering that their large size is owed to large introns and not to the coding regions of the genes. This suggests that CFSs might have a physiological role in the organisms. So far there are indications pointing to a possible role of CFS-associated replication stress in neuronal diversification, however further investigation is needed to establish whether that is true or not. Future studies focusing on the role of the large introns of CFS genes both on cellular and organismal level will be necessary. Identifying the potential physiological role that replication stress might have in an organism is key to understand why CFSs are conserved and it will pave the way for a better understanding of cancer, brain development, and neurodegenerative diseases.
